# An Ayurgenomics Approach: Prakriti-Based Drug Discovery and Development for Personalized Care

**DOI:** 10.3389/fphar.2022.866827

**Published:** 2022-04-01

**Authors:** Zoufang Huang, Vivek P. Chavda, Rajashri Bezbaruah, Vladimir N. Uversky, Sucharitha P., Aayushi B. Patel, Zhe-Sheng Chen

**Affiliations:** ^1^ Ganzhou Key Laboratory of Hematology, Department of Hematology, The First Affiliated Hospital of Gannan Medical University, Ganzhou, China; ^2^ Department of Pharmaceutics and Pharmaceutical Technology, L M College of Pharmacy, Ahmedabad, India; ^3^ Department of Pharmaceutical Sciences, Faculty of Science and Engineering, Dibrugarh University, Dibrugarh, India; ^4^ Department of Molecular Medicine and Byrd Alzheimer’s Research Institute, Morsani College of Medicine, University of South Florida, Tampa, FL, United States; ^5^ Department of Pharmaceutics, Seven Hills College of Pharmacy, Tirupati, India; ^6^ Pharmacy Section, L M College of Pharmacy, Ahmedabad, India; ^7^ Department of Pharmaceutical Sciences, College of Pharmacy and Health Sciences, St. John’s University, Queens, NY, United States

**Keywords:** ayurgenomics, genomics, ayurveda, diet, lifestyle, disease, pharmacogenomics

## Abstract

Originating in ancient India, Ayurveda is an alternative medicinal approach that provides substantial evidence for a theoretical-level analysis of all aspects of life. Unlike modern medicine, Ayurveda is based upon tridoshas (*Vata, pitta, and Kapha*) and *Prakriti.* On the other hand, the research of all the genes involved at the proteomics, metabolomics, and transcriptome levels are referred to as *genomics*. Geoclimatic regions (deshanupatini), familial characteristics (kulanupatini), and ethnicity (jatiprasakta) have all been shown to affect phenotypic variability. The combination of genomics with Ayurveda known as ayurgenomics provided new insights into tridosha that may pave the way for precision medicine (personalized medicine). Through successful coordination of “omics,” *Prakriti*-based treatments can help change the existing situation in health care. *Prakriti* refers to an individual’s behavioral trait, which is established at the moment of birth and cannot be fully altered during one’s existence. Ayurvedic methodologies are based on three *Prakriti* aspects: *aushadhi* (medication), *vihara* (lifestyle), and *ahara* (diet). A foundation of *Prakriti*-based medicine, preventative medicine, and improvement of life quality with longevity can be accomplished through these ayurvedic characteristics. In this perspective, we try to understand prakriti’s use in personalized medicine, and how to integrate it with programs for drug development and discovery.

## Introduction

“Ayurveda is an extensive, natural system of health care that originated in ancient India during the Vedic period (Pandey et al., 2013).” The word “Ayurveda” originates from Sanskrit: “*Ayus*,” which means lifespan or life, and “*Veda*,” which suggests science or knowledge. Taken together, Ayurveda means “the science of life,” or “the science of lifespan.” Unfortunately, many Ayurveda and *Veda* concepts are not well-defined from the perspective of existing knowledge, which results in conflicting interpretations of current communication (Sharma and Keith Wallace, 2020). After several years of foreign conquests of India, information became scattered and forgotten.

Wallace writes, “The *Tridosha* theory of Ayurveda explains that there are three fundamental principles or forces, called *doshas*, which govern the physiology of each individual.” Each *dosha* has its own set of characteristics and functions. *Vata* is a *dosha* responsible for transfer inside the body which aids in the transmission of molecules to nerve impulses (Sumantran and Tillu, 2013; Prasher et al., 2017). *Vata* adds to shape manifestation, cell division, signaling, waste elimination, movement, thought, and it also normalizes the function of *Pitta* and *Kapha. Pitta* regulates digestion and various metabolic processes within a cell. It also is responsible for energy balance, thermoregulation, vision, pigmentation, and host surveillance, among other things (Prasher et al., 2016). The *dosha* of *Kapha* oversees the body’s structure and cohesiveness, including storage, stability, development, and maintenance. *Prakriti* is the mix of these three *doshas* that each human is born with (Wallace, 2020). The *Tridoshas* are made up of all five *Mahabhutas* [Air (*Vayu*), Water (*Jal*), Fire (*Agni*), Earth (*bhumi*), and Space (*Aakash*)], but one or the other is dominating, with the other four having a smaller influence (Sumantran and Herbal, 2011). There can never be a condition in which one or both *Mahabhutas* are completely missing. All five are required for life to exist. A proper balance of these three *Doshas* is necessary for optimal health (Rotti et al., 2014a). *Vata Dosha* is made up of the *Mahabhutas Akasa* (space) and *Vayu* (air). *Tejas or Agni* (fire) and Ap (water) *Mahabhutas* make up *Pitta Dosha*. Ap (water) and Prithvi (earth) *Mahabhutas* make up *Kapha Dosha*. When there is a *Dosha* imbalance, one says that a person is *Vata, Pitta,* or *Kapha* dominant. Each *Dosha* imparts certain characteristics to the individual, based on which an individual can be categorized as belonging to that specific *Dosha* type (Shilpa and Venkatesha Murthy, 2011). According to *Charaka* and *Sushruta*, persons may be categorized into seven sorts or groups based on the prevalence of the *Doshas* in their bodies. They are considered to belong to a specific *Prakriti* or constitution in the following ways: People who have a prominent *Vata Dosha*, also known as *Vata Prakriti* (or constitution). There are people who have a predominant *Pitta Dosha/Prakriti*, with a strong *Kapha Dosha/Prakriti*, with a predominant *Vata–Pitta Dosha* combination/*Prakriti*, with a strong *Vata–Kapha Dosha* combination/*Prakriti*, with a prominent *Pitta–Kapha Dosha* combination/*Prakriti*, with balanced *Doshas* or a mix of *Vata–Pitta–Kapha Doshas* (Rotti et al., 2014a; Gadre, 2019). Each person is born with a unique percentage of *tridosha* that is determined not just by genetics but also by the milieu throughout fetal development. As the characteristics of individual are defined on the basis of *Dosha* predominant Prakriti and the *Dosha* dominance regulates the physiology of the individual, defining preventive and treatment on this basis may help to identify the life style intervention along with medicines for its best effect. In *Dashavidha Pariksha*, which is the basis of personalized medicine, consideration of *Dosha Prakrtiti* has been done (*Charaka Samhita*).

According to Gupta PD (Gupta, 2015), “Perturbation of the *tridoshas* in an individual from his or her homeostatic state leads to diseases. These proportions of *tridoshas* are determined genetically (*Shukra* short) and are influenced by the environment (maternal diet, lifestyle) during development.” However, culture, ancestral features, and location of origin have an impact on *Prakriti.* The onset of certain doshic illnesses occurs when *Vata, Pitta,* and *Kapha* are increased past an individual’s threshold.

*Prakriti* relates to the *dosha* balance at conception, whereas *Vikruti* alludes to the *dosha* balance in the present, and hence identifies the kind of imbalance or sickness. *Prakriti* and *Vikruti* are both made up of several “*Doshas*” in different quantities (Wallace, 2020). Ayurveda emphasizes whole-body therapy by integrating physical, emotional, and mental health and believes that a person’s *dosha*, or physiological humor, affects their character and wellbeing (Prasher et al., 2016). Amongst vatic disorders described are, neurological, developmental, speech, and motion disorders, dementia, arrhythmias, and so on. Conditions such as ulcers, skin diseases, bleeding disorders, etc. are described in *Pitta* elevation, while diabetes, obesity, and atherosclerotic conditions are described in *Kapha* elevation. The disturbances of particular *doshas* in a person are evaluated through indications, and the goal inherited with ayurvedic physicians is the quantification of the amount of disturbance followed by its restoration to their homeostatic state *via* proper dietary and therapeutic regimes (Prasher et al., 2016). All foods, drugs, and lifestyle-related objects have been identified as augmenting or mitigating a specific *doshic* state, resulting in a tailored and specific therapy. Thereby, the elegance of Ayurveda derives from the statistic that an individual, an ailment condition, medication, food, and the situation are all presented in the way of doshic elements, and appropriate customizations will be delivered to stabilize these states. Even though *Prakriti* and tridoshas encapsulate foundations of personalized ayurvedic precepts for implementation in prognostic treatments, it is crucial to create their molecular basis (Haider et al., 2021).

A genome is a complete set of DNA that includes all of an organism’s genes along with its hierarchical, three-dimensional structural configuration. The human genome is made up of 23 chromosomal pairs (diploid), each with 3 billion base pairs of DNA inherited from either parent. There are several variants in the human genome sequence known as single nucleotide polymorphisms (SNP). Some of these differences are frequent and seen in a significant number of individuals, whereas others are uncommon. If the differences occur in fewer than 1% of the population, they are most likely categorized as mutations. Many uncommon disorders are monogenic, meaning they are caused by mutations in a single gene, for example, hemophilia and beta-thalassemia. However, diabetes, asthma, and cardiovascular disease are multigenic complicated disorders, i.e., including many genes (Aggarwal et al., 2010). Genomics is a branch of biology that focuses on the evolution, function, structure, editing, or mapping of genomes (Seehausen et al., 2014). Basically, genomics is a predictive personalized interpretation of what DNA tells about the respective individual based on genetic makeup and Ayurveda is an ancient personalized medicinal system, where the appropriate drug and dietary regime are chosen based on a clinical examination of the patient’s disease endophenotype, basic constitution, and health status at the time of administration (Sethi et al., 2011). The interaction of genetic networks and environmental factors is important for phenotypic diversity in health and disease. Every person has his/her genetic make-up, i.e., *Prakriti* (Bhadresha et al., 2020). Scientists at CSIR-Institute of Genomics and Integrative Biology (CSIR-IGIB) in New Delhi, The ministry of science and technology, hypothesize an opportunity to analyze if the *Prakriti*-based characterization of individuals has a genetic basis (Prasher et al., 2022). For the first time, assimilation of ayurvedic *Prakriti,* classification methods with modern genomics resulted in the discovery of the molecular and genomic basis of the theory of *Dosha Prakriti*. Healthy people of different *Prakriti* types, as recognized by Ayurveda, display significant differences in biochemical and hematological parameters. Genomic studies showed that the main *Prakriti* types differed significantly in gene expression levels, particularly those involved in immunity, cell division, blood coagulation, and so on (Chatterjee and Pancholi, 2011).

Ayurveda and genomics can benefit from each other, especially *via* preventive care strategies. In particular, it is certainly relevant for the P4 medicine (four Ps are predictive, preventive, personalized, and participatory), which is rooted in some of the ayurvedic fundamentals (Flores et al., 2013; Lemonnier et al., 2017). Individuals would be able to engage in self-care more easily if they had access to time-tested personalized prevention and lifestyle control suggestions. For the first time, the incorporation of ayurvedic *Prakriti* differentiation approaches with advanced genomics enabled the identification of the molecular genetic basis of the concept of *Dosha Prakriti* (Hawkins et al., 2010; Chatterjee and Pancholi, 2011; Prasher et al., 2016).

**FIGURE 1:**
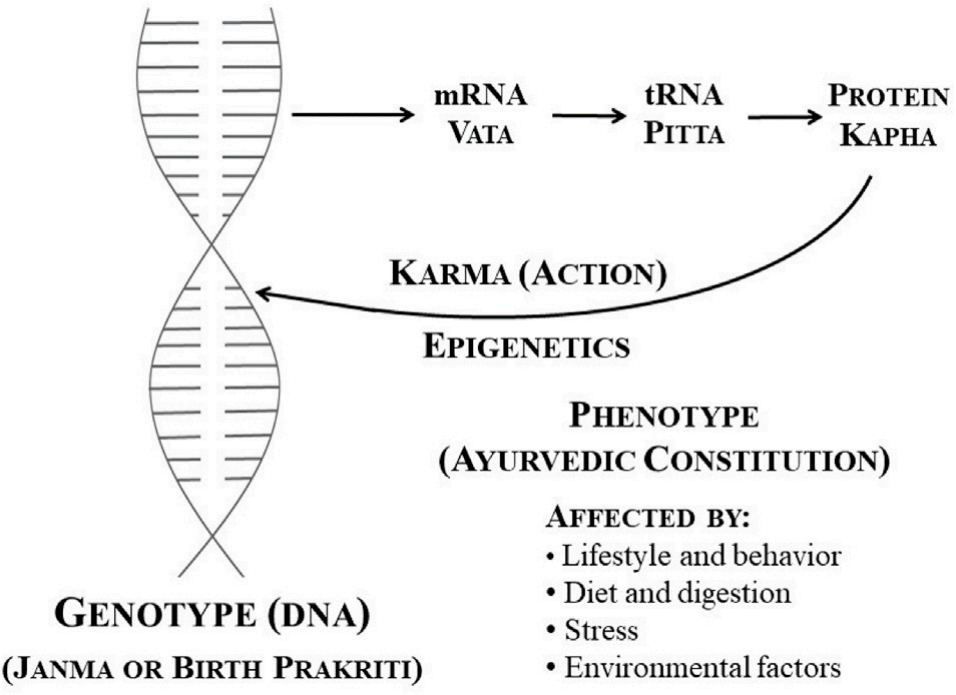
DNA and cellular activity, as well as their relationship to Ayurveda [Adopted from (Sharma and Keith Wallace, 2020) under CC BY 4.0 Licence].

Ayurgenomics appears to be based on the same principles as pharmacogenomics/pharmacogenetics and can function as a framework for realizing the theory of personalized drug treatment (Figure 1). Some initiatives have been established to include Ayurveda with pharmacogenetics, such as the incorporation of epigenetics with Ayurveda, ayurnutrigenomics, and so on. Ayurgenomics is now generating a lot of research attention in the area of personalized drug development. In this light, *via* this review, we want to comprehend *prakriti’s* use in personalized medicine, as well as how to combine it with drug development and discovery initiatives. Stratification will help better understand the normal phenotypic variations. If we classify the population without *prakriti* type and go by the biomarker-based approach; a large sample size is required to prove it statistically significant. secondly, the enormous heterogeneity in the disease state may mask the true effect. The multiscale nature of the genome-wide association is nearly intractable to approaches generally undertaken.

## Epigenetics in Ayurveda

The study comprising the determination of the effect of environmental and behavioral changes on gene functioning is termed epigenetics. Epigenetics states the peripheral change of DNA, which switches genes on and off thereby inducing gene expression. In contrary to genetic changes, changes in epigenetics are alterable, and they will not alter the DNA sequence, but they can alter how the body recognizes a DNA sequence (Bird, 2007; Cheng et al., 2019). Such kind of process results in a phenotypic change without genotypic change. The genotype is the aspect of a person’s genetic makeup that determines particular characteristics (coding genes). Until there is any toxic damage, a genotype is not changing. Whereas an individual’s phenotype such as appearance, behavior, and development is dependent on genotype. However, some environmental factors lifestyle or psychological factors may affect an individual’s phenotype, which accordingly may alter the genotype expression (Jelenkovic et al., 2016; Sharma and Keith Wallace, 2020).

Epigenetics is believed to be a key component of Ayurveda, as it amalgamates the genotypic and phenotypic variations. Ayurveda describes the factors (behavior, lifestyle, stress, diet, and digestion, as well as the environment) influencing the *Deha* (body) *Prakriti* (psychological and physiological composition), which relates to the phenotype, along with *Janma* (birth) *Prakriti,* which relates to the genotype. Understanding this will result in improved interaction and comprehension with the existing healthcare system, and greater connectivity of both fields of science in the operations of good health (Sharma and Keith Wallace, 2020). *Prakriti*, which is fixed at birth, remains constant throughout one’s life. This is independent of ethnic, racial, or geographical factors, and can include a suitable method of categorizing phenotypes for genotyping. As a result, it categorizes medicine in the following ways called Rasapanchaka (ayurvedic pharmacology), which indicates that a drug’s activity is related to positive drug attributes such as Prabhava (effect), Guna (property), Virya (potency), Rasa (taste), and Vipaka (post-digestive taste), whereas modern pharmacology categorizes drug activity based on molecular chemical structure (Sethi et al., 2011; Gupta, 2015).

*Vikriti* is an asymmetry or disease of the *Deha Prakriti* that relates to disorders in humans in the modern medical system. At the molecular level, it is suggested that tRNA, proteins, and mRNAs have properties and characteristics that reflect the three ayurvedic *doshas—Kapha, Pitta,* and *Vata* (Prasher et al., 2008; Sharma, 2016; Sharma and Keith Wallace, 2020)*.* Four significant factors affect the phenotype or *Deha Prakriti*, either positively or negatively, based on an individual’s actions in life: 1) behavior and lifestyle; 2) digestion and diet; 3) stress 4) environmental factors (Prasher et al., 2008). Ayurveda describes these four main aspects of life, positively influencing both the genotype and phenotype *via* the epigenetic process. As a result, it is suggested that epigenetics is a critical component in Ayurveda (Storey et al., 2007; Banerjee et al., 2015). Future generations may be impacted by epigenetic changes. Nutritional deficiencies, environmental toxins, and environmental stress can all produce epigenetic changes that are passed down to future generations, resulting in illness (Shilpa and Venkatesha Murthy, 2011). Exposure to air pollution during pregnancy has been demonstrated to alter the gestating mother, her embryo, and its growing germ line, affecting the phenotypic of the third generation (Sharma and Keith Wallace, 2020).

Both *Prakriti*-oriented medicine and customized medicine emphasize the significance of health enhancement and illness management (Juyal et al., 2012). The domain of epigenomics examines the impact of proteins, metabolic processes, genetic and non-genetic variables on human physiology, as well as differences in mechanisms that play a crucial part in an individual’s health risk. Ayurgenomics can help explain how to present medications can be utilized more successfully if they are targeted at people with certain Prakriti (Chatterjee and Pancholi, 2011)*.*

## Ayurnutrigenomics

According to Ayurveda, the concepts of food and medicine are intertwined and are crucial for human self-preservation as well as disease management and mitigation. Food disrupts the biomolecular basis of a creature’s “physiome.” It is eaten up in greater quantities than any other drug. As a result, examine into its effect on and interplay with the genome was most important for understanding illnesses and their treatments (Banerjee et al., 2015). Ayurgenomics is a tailored methodology in curative, preventive along with predictive facets of stratified drug with biochemical changeability that encapsulates the research of inter-individual variation caused by differences in genetics in living beings for evaluating vulnerability and constructing prognosis and diagnosis, primarily based on a person’s *Prakriti* constitution type (Sethi et al., 2011; Banerjee et al., 2015).

“Ayurnutrigenomics is an emerging field of interest pervading Ayurveda systems biology, where the selection of a suitable dietary, therapeutic, and style of living is made based on clinical assessment of an individual maintaining one’s *Prakriti.* This Ayurveda-inspired concept of personalized nutrition is a novel concept of nutrigenomic research for developing personalized functional foods and nutraceuticals suitable for one’s genetic makeup with the help of Ayurveda (Prasher et al., 2016).” The exploratory study conducted by Juyal and colleagues suggests, “Discrete causal pathways for rheumatoid arthritis etiology in Prakriti based subgroups, thereby, validating concepts of *Prakriti* and personalized medicine in Ayurveda. Ayurgenomics approach holds promise for biomarker discovery in complex diseases (Juyal et al., 2012).” Ayurveda provides its methodologies through the three main pillars of *Prakriti*-based healthcare: *Aushadhi* (medication), *Vihara* (lifestyle), and *Ahara* (diet) (Juyal et al., 2012). The foundation of personalized medicine, disease control, and improvement from health to strength and durability with a better standard of living, could be accomplished through these ayurvedic attributes (Amin et al., 2017). Prakriti has the potential to overcome the existing curb of clinical heterogeneity in biomolecular, genetic investigation of structural characters. It can be used to categorize diseased as well as healthy people. Unfortunately, the central issue of ayurgenomics may be defining a link between DNA and *Prakriti* (Govindaraj et al., 2015).

## Research Notes on Ayurgenomics and Drug Discovery and Development for Personalized Care

Over a decade ago, a link between the concept of *Prakriti* and genetics was proposed. Several serious attempts have been made to link *Prakriti* categorization with genetic information and the association of SNPs in order to verify the genetic and molecular foundation of *Prakriti* (Sharma and Prajapati, 2020). Mitali Mukerji is a Chief Scientist at the CSIR Institute of Genetics and Integrative Biology has made noteworthy contributions to personalized medicine and human genomics (Vianu, 2015). A detailed investigation linking biological and genome-wide levels of expression in participants among the three main *Prakriti* groups was published in 2008 by her team. The participants in the study were normal healthy people from the three extreme and differing *Prakriti* groups—*Vata, Pitta,* and *Kapha*. The CYP2C19 gene was studied, and it was discovered that there is a strong link between this gene and the *Prakriti* phenotype. The research reveals that there was an over-expression of genes in the immune response pathways in *Pitta* dominants. *Kapha* males had higher levels of metabolic syndrome and chronic inflammation markers than *Vata* males, which was consistent with over-expression of genes involved in inflammatory response in these individuals. Prothrombin time, an indicator of blood coagulation, was found to be low in *Kapha* males. Besides that, increased levels of haemoglobin gene expression in *Pitta* compared to *Vata* and *Kapha* corroborate the differences in haemoglobin levels between the *Prakritis* and coincide with skin redness as a phenotype in *Pitta* individuals. Genes involved in fibrinolysis were down-regulated in *Kapha Prakriti* men, while genes involved in ATP and cofactor production were up-regulated. Furthermore, the researchers discovered that *Kapha* types have greater levels of total cholesterol, triglycerides, high-density lipoprotein (HDL), and low-density lipoprotein (LDL) than *Pitta* and *Vata* kinds (Prasher et al., 2008). Our review of the literature found that a variety of prakriti diagnostic methods have been used in diverse studies, but only a few of them have been standardized (Rastogi and Chiappelli, 2012; Rotti et al., 2014b).

In a study in 2005, 76 people were tested for their *Prakriti* types against 14 different alleles of human leucocyte antigen (HLA) DRB1 and found that the allele frequency varies depending upon *Prakriti* types. The study found a link between HLA type and *Prakriti* type, with the *Vata* type lacking the HLA DRB1*02 allele and the *Kapha* type lacking the HLA DRB1*13 allele. Likewise, HLA DRB1*10 allele frequency was higher in the *Kapha* type than in the *Pitta* and *Vata* types (Bhushan et al., 2005).

In 2010, Aggarwal et al. (2010), analyzed the connection of the EGLN1 gene [frequent variants rs479200 (C/T) and rs480902 (T/C)] in high-altitude adaptation. They also discovered the TT genotype of rs479200 which was more common in *Kapha* types and was linked to greater EGLN1 expression. In *Pitta*, on the other hand, it was far less common and almost non-existent in high-altitude locals (Aggarwal et al., 2010).

Furthermore, Ghodke et al. (2011), exposed that CYP2C19 genotypes, a family of genes that are associated with the metabolism and detoxification of all the drugs, were down-regulated in *Kapha* types and up-regulated in *Pitta* types. A research study conducted in 2012 found that *Kapha* types have greater levels of natural killer cells (CD56) activated B cells (CD25) (Rotti et al., 2014a). Inflammatory genes were up-regulated in *Vata* types, whereas protein sequences involved in the oxidative stress pathway were up-regulated in *Pitta* and *Kapha* types, according to RC Juyal and colleagues (Juyal et al., 2012). One of the most comprehensive types of research published in 2015 discovered that 52 SNPs were pointedly dissimilar in the three primary forms of *Prakriti*. SNPs (pronounced “snips”), or single nucleotide polymorphisms, are the most prevalent form of genetic variation seen in humans. Each SNP indicates a change in a single nucleotide, which is a type of DNA-building component. It also presented that the SNP in the PGM1 gene (rs11208257) is likewise linked to energy generation and is additionally homogeneous and consistent in *Pitta* body types than in *Kapha* and *Vata* types (Govindaraj et al., 2015). PGM1 (Phosphoglucomutase 1) is a Protein Coding gene and an evolutionarily conserved enzyme that governs one of the most significant metabolic carbohydrate trafficking sites in prokaryotic and eukaryotic organisms, facilitating the bi-directional interconversion of glucose 1-phosphate (G-1-P) and glucose 6-phosphate (G-6-P) (Wallace, 2020; Gururaj et al., 2004). G-1-P generated by sucrose catabolism is transformed to G-6-P, the first intermediate in glycolysis, in one way. PGM1 coincides with the *Pitta* phenotype as described in the ancient literature *Charaka Samhita*, implying that the phenotypic classification of India’s traditional medicine has a genetic foundation (Prasher et al., 2008; Govindaraj et al., 2015; Rotti et al., 2015); and its *Prakriti*-based treatment, which has been popular for ages, resonates with personalized medicine (Govindaraj et al., 2015). Around 3,416 healthy male candidates with diverse ethnic and linguistic groups and inhabited different geographical regions were enrolled with monitoring of their health status with “AyuSoft” Software (http://ayusoft.cdac.in). Leading Ayurvedic physicians calculated the content of *Prakriti*, which was rigorously validated by “AyuSoft,” a program built based on historical Ayurvedic literature (Bhargav et al., 2021). According to them, “52 SNPs (*p* ≤ 1 × 10−^5^) were significantly different between *Prakriti*, without any confounding effect of stratification, after 106 permutations. The principal component analysis (PCA) of these SNPs classified 262 individuals into their respective groups (*Vata, Pitta*, and *Kapha*) irrespective of their ancestry, which represents its power in categorization. They further validated their finding with 297 Indian population samples with known ancestry. Subsequently, They found that PGM1 correlates with the phenotype of *Pitta* as described in the ancient text of *Charaka Samhita*, suggesting that the phenotypic classification of India’s traditional medicine has a genetic basis; and its *Prakriti*-based practice in vogue for many centuries resonates with personalized medicine.”

A 2015 study discovered DNA methylation markers that differentiate types of *Prakriti.* The study speculates that DNA methylation is likely linked to the regulation of chromatin as a donor to distinct *Prakriti* phenotypes and by this way, this research sheds light on the ayurvedic individualized system of medicine’s epigenetic mechanisms (Rotti et al., 2015). Recent work on *Cissampelos pareira L.* transcriptome investigation and connection mapping found molecular linkages between ESR1 alteration and viral suppression (Haider et al., 2021).

**FIGURE 2:**
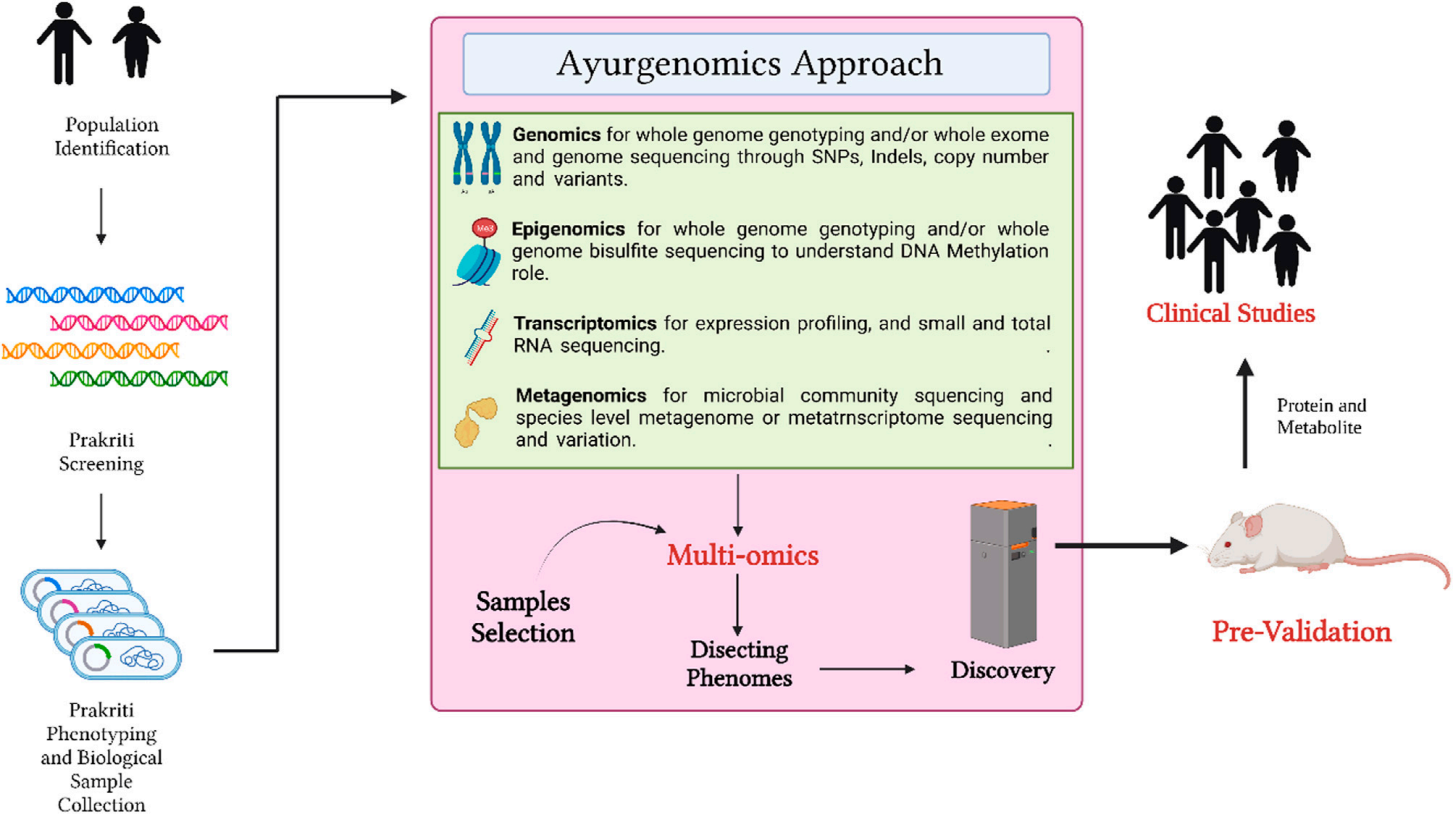
Ayurgenomics approach for drug discovery towards personalized medicines.

In addition to the research studies, there have been several reviews as well as theoretical papers were publishing promoting the concept of ayurgenomics. Moreover, few articles were published focusing on some related fields like ayurnutrigenomics, pharmacogenetics, etc. (Bhat et al., 2021). The review suggested that research on the *Prakriti-*based categorization of genetic differences, distinct immunological response, and clinical aspects of COVID-19 would better determine forecast and develop an effective therapy plan. As a result, Ayurveda-based phenotyping might provide an efficient and comprehensive diagnostic technique for COVID-19 incident management, control, and individualized care (Figure 2) (Amin et al., 2017).

For centuries, Ayurveda has been a source of natural or plant-based medicine in many drug development efforts, although with the hunt for novel chemical entities (Patwardhan and Mashelkar, 2009). Many scientific articles demonstrate the efficacy and promise of Ayurvedic medicines. For example, during the current Covid-19 pandemic, an in-silico evaluation on AYUSH-64 (an Ayurvedic formulation produced and patented by India’s Central Council of Research in Ayurvedic Sciences that has been in clinical use for several decades as an anti-malarial, anti-inflammatory, and anti-pyretic medication) showed that the medicine was a promising candidate for repurposing to COVID-19 treatment (Ram et al., 2022). Additionally, some inimitable insights have been found following systematic investigation of the biological effects of herbs. For example, one of the most notable ayurvedic medicament “reserpine” (active constituent of the herb “Sarpagandha”) allowed the dissection of the complete dopaminergic pathways and the fusion of psychopharmacology and neurochemistry (Sanjeev Jain, 2009). Ayurveda, in addition to its curative capabilities, provides a predictive, preventative, and personalized strategy to health and illness management that has been thoroughly described in classic literature such as the Charaka and Sushruta Samhita (Patwardhan et al., 2014; Banerjee et al., 2015). This concept paves the way for ayuregenomics drug development.

The CSIR Ayurgenomics Unit, TRISUTRA has taken collaborative endeavor across ethnically and geographically varied Indian communities to develop ayurgenomics for stratified medicine. The study entails the selection of study cohorts from five geographical sites where healthy individuals are enrolled for ayurgenomics studies through community engagement. Before the screening of *Prakriti*, the subject’s health is assessed utilizing a short questionnaire after the subject has given his or her agreement to participate in the study. Oral saliva is collected for DNA isolation throughout the screening process. A subset of predominant and mixed Prakriti individuals is recalled for detailed clinical phenotyping and sample collection after a preliminary *Prakriti* examination. Various anatomical variables relating to body structure and composition, such as length, shape, size, symmetry, and breadths, as well as bulk and quality of musculature of different individual body parts, are considered in the Prakriti assessment. Nevertheless, *Prakriti* types differ in terms of different skin characteristics such as look, color, nature, and texture. Heart rate, vital capacity, taste threshold assessments were also considered. Aside from extensive *Prakriti* assessments, objective examinations of various anatomical components and systems are carried out. Blood, feces, and urine samples from the periphery are collected and processed for biochemical, molecular, and omics analysis later on. The participants undergoing thorough phenotyping have their peripheral blood, stool, and urine collected. DNA is isolated from saliva samples, whereas DNA, RNA, serum, and plasma, as well as blood cells, are isolated from peripheral blood samples. For metagenomic analysis, DNA is also extracted from the stool and oral saliva. For metabolite analysis, urine samples are dried and stored on filter sheets. Then leads are tested using different model systems for cellular, physiological or phenotypic outcomes. Functional validation of these findings would be required to translate genome-scale maps of genomic and epigenomic markers to clinically useful information. Validation of *Prakriti* findings is also done in *Prakriti*-based LCL lines and PBMCs utilizing well-established methods and *Dosha*-specific treatment regimens (Prasher et al., 2017). The entire procedure is depicted in Figure 2. The preclinical studies help to findout the basis of drug discovery in clinical trials. Animal models use to validate the scientific approach and correlation with the further tragets in clinical trials. Many fundamental principles of Ayurveda proven through animal studies with correletaion of many objective and subjective parameters (Payyappallimana and Venkatasubramanian, 2016; Rao, 2018).

## Conclusion and Future Perspectives

Over 65% of Indians follow “Ayurveda,” an indigenous Indian system of medicine that is growing generally recognized as an alternative medicine across the globe. Ayurveda follows a personalized strategy for prognostic and preventive elements of medicine. It relates to the inter-individual heterogeneity in vulnerability assessment, and judgment, commonly based on the individual’s *Prakriti* types. Modern medicine employs terminologies like gene expression, genome, epigenetics, etc. to define the foundation of our physiology and health in a profoundly reductionist framework. While Ayurveda uses a unique holistic approach that encompasses concepts such as *Prakriti* and *doshas*. Because of a mental bias contrary to folk or traditional medicine, modern medicine has failed to acknowledge many of Ayurveda’s effective preventative techniques. Even though traditional medical systems are in still exist and widely practiced in many nations and around the globe, further research into their preventative and rehabilitative treatment programs is required. To date, the focus of the study has been on certain herbal formulations, to isolate a single drug moiety that must subsequently be employed by the pharma industry.

Ayurgenomics was established only to understand the principles of Ayurveda using the most recent contemporary methods, paving the door for evidence-based Ayurveda and therefore a greater worldwide recognition. Ayurgenomics creates a new-fangled link between modern and traditional medicine by offering a scientific grasp of fundamental ideas while also infusing Ayurveda’s practical prophylactic techniques into modern care. This revolutionary system intends to change the focus from a disease-focused system to a patient-focused wellness system. It is strongly connected to other emerging areas such as personalized, integrative, preventive, lifestyle, and functional medicine. Even before epigenetics and other studies like nutrigenetics, Ayurveda understood how nutrition and other lifestyle variables might influence human health. They documented that contemporary medicine is only now a foundation to recognize: anticipation is vital for good health. An efficient preventative medical system requires proper exercise, sleep, food, and stress management. Additionally, ayurveda also explains the dose and duration of the drug, which is determined by the patient’s bala, satva, agni, roga bala, and so on. Ayurgenomics helps to increase the legitimacy of Ayurveda and other traditional medicinal systems by expressing their old concepts in terms of current science. Emerging ayurgenomics techniques that employ powerful data analytics and machine learning might greatly simplify the procedure. Ayurgenomics’ medicinal efficacy will have to be tested in strictly managed clinical studies. Ayurveda and genetics have a lot to learn from one another. Contemporary science, being an evidence-based medical system, may help Ayurveda, and Ayurveda must uplift modern medicine, predominantly through its pre-emptive measures.

Researchers hoped that rapid advances in genetics would lead to a personalized medical system that could forecast and avoid disease. The ultimate objective of ayurgenomics is to achieve personalized medicine. Personalized medicine is the use of diagnostic and screening procedures to better treat an illness or propensity to a disease in an individual patient. The foundation of personalized medicine is constitute with the combination of full gene mapping and drug response studies, which permit to achieve the aim of providing the appropriate medication to the right patient at the right time.

Although advancement has been achieved, due to the extremely complicated nature of gene expression in the progress of illness states, the complete development of the concept of ayurgenomics may take longer than expected. Patients’ compliance with disease care is a primary issue in the rapidly expanding era of genomic and molecular medicine, as is the need for medications to work better with fewer side effects to assure greater health. Physicians must use treatment alternatives that result in more accurate treatment and lower the chance of diagnostic errors. Given the molecular genetics disparities underpinning *Prakriti,* and its importance in disease pharmacogenomics research, this innovative integrative system of ayrgenomics could assist in the diagnosis of differentially vulnerable and drug-responsive populations. Furthermore, a combined assessment of genomic and phenomic variability may enable the identification of biomedical and epigenetic indicators of *Prakriti* to use in tailored medicine, as well as its incorporation into drug development and discovery programs. Many people believed that the tremendous development in genomics will lead to a personalized medicine system that might forecast and prevent illness. Based on available research and theoretical data, we can assume that; the merging of Ayurveda and genomics has the potential to significantly enhance many aspects of global healthcare. Ayurgenomics construe the fundamental principles and individual therapy of Ayurveda with the objective tools in modern sciences and therby understanding and development of evidence based therapy for its global acceptance. If scientists are successful in demonstrating molecular proof that various Prakriti have varied baseline thresholds, we will be able to establish economical health care solutions through early screening in community health programmes for early treatments. Furthermore, identifying biological markers that correspond with the outcome of Ayurvedic therapeutic procedures such as panchkarma will expand the acceptance and accessibility of such therapies.
